# Non-adherence to anti-tuberculosis treatment, reasons and associated factors among TB patients attending at Gondar town health centers, Northwest Ethiopia

**DOI:** 10.1186/s13104-018-3789-4

**Published:** 2018-10-01

**Authors:** Habtamu Sewunet Mekonnen, Abere Woretaw Azagew

**Affiliations:** Department of Medical Nursing, School of Nursing, College of Medicine and Health Sciences, University of Gondar, Gondar, Ethiopia

**Keywords:** Prevalence, Reasons, Non-adherence, Tuberculosis treatment, Ethiopia

## Abstract

**Objective:**

The aim of this study was to assess the prevalence of non-adherence to anti-tuberculosis treatment, reasons and associated factors among TB patients attending at Gondar town health centers.

**Result:**

A total of 314 participants were included with the response rate of 97.5%. The mean age of participants was 35.94 (SD ± 13.83) years. The overall rate of non-adherence to anti-TB treatment was 21.2% (95% CI 17.2, 26.1). Continuation phase of treatment (AOR = 2.27, 95% CI (1.54, 5.94)), presence of more than one co-morbidity (AOR = 6.22; 95% CI (2.21, 17.48)), poor knowledge about TB and anti-TB therapy (AOR = 4.11; 95% CI 1.57, 10.75), poor patient-provider relationship (AOR = 4.60, 95% CI 1.63, 12.97), and alcohol intake (AOR = 5.03; 95% CI 1.54, 16.40) were significantly associated with non-adherence. Forgetting 40 (23.1%), Being busy with other work 35 (20.2%), and being out of home/town 24 (13.9%) were the major reasons of participants for interruption of taking anti-TB medications.

**Electronic supplementary material:**

The online version of this article (10.1186/s13104-018-3789-4) contains supplementary material, which is available to authorized users.

## Introduction

Tuberculosis (TB) is airborne infectious disease caused by *Mycobacterium tuberculosis* [1]. It is one of the ten top causes of death worldwide from curable infectious diseases. Globally there were estimated 10.4 million new TB cases, and 600,000 new cases with resistance to rifampicin, 490,000 had multidrug-resistant tuberculosis (MDR-TB) cases and 1.7 million people died from TB [1–3].

The main risk factors for developing active TB case are human immunodeficiency syndrome (HIV) infection, low socioeconomic status/poverty, alcoholism, homelessness, crowded living condition, diseases that weaken the immune system, migration from country with high number of cases, and health-care workers [4]. Tuberculosis non-adherence is the major challenge in TB treatment which leads multidrug as well as extended drug-resistant TB [5, 6]. Combating non-adherence is the key and cornerstone of anti TB treatment. The prevalence of non-adherence to anti-tuberculosis treatment is 50% India, 15.5% Thailand, 24.7% and 24.5 South Ethiopia [7–10].

The main reasons for non-adherence in anti-tuberculosis treatment are drug side effects, forgetting to take medication, be away from home, missing date of appointment, lack of transportation cost, lack of social support, poor communication between patient and healthcare providers, and stock out of medicines [11–13]. Non-adherence to anti TB treatment results in increased length and severity of illness, death, disease transmission and drug resistance. It has great economic impact in terms of cost to patients as well as the health care system [13, 14].

Adherence to long course TB treatment is complex, dynamic phenomenon with wide range of factors impacting on treatment taking behaviors [15]. Even though there is wide coverage of DOTs program in Ethiopia, there is paucity of evidence on rate, reasons and associated factors of non-adherence on anti TB treatment particularly in the study area. Therefore the present study determines prevalence, reasons and associated factors of non-adherence of anti TB treatment among TB patients.

## Main text

### Study design and setting

Institutional based cross-sectional study design was conducted among TB patients from May to June 2017 at Gondar town health centers. Gondar town is found about 737-km away from Addis Ababa. In the town, there are one governmental specialized hospital, one private hospital and eight governmental health centers which serve for more than five million populations. Maraki, Polly, and Azezo health centers were selected. In these health centers, there were around 713 TB patients.

### Source population

All TB patients who were on anti TB treatment in Gondar town health centers were considered as source population.

### Inclusion/exclusion criteria

All TB patients who took anti TB medication at least for 1 month were included in the study, whereas TB patients who were seriously ill and or unable to hear and speak were excluded.

### Sample size and sampling procedure

The sample size was determined using single population proportion formula (n = [(Zα/2)2 × P (1 − P)]/D2) with the assumption of 95% level of confidence and 5% marginal error. Prevalence of non-adherence to anti TB treatment was taken 24.5% [10]. Taking 10% nonresponse rate the required sample size was 314. Using simple random sampling three health centers were selected. Proportional allocation method TB patients were taken from selected health centers (Maraki health center 88, Polly health center 110, and Azezo health center 116) then each study participant was selected using systematic random sampling. Sampling interval (K) was ~ 3 in each health center thus every three participants were interviewed based on their order of arrival.

### Data collection tools and procedures

Data was collected by using pretested structured questionnaire adapted from different literatures [10–12]. The questionnaire has socio-demographic information, characteristics of tuberculoses and anti- tuberculoses treatment, reasons for interruption taking medications, knowledge and attitude towards tuberculosis and anti-TB treatment, patient-provider relationship, and behavioral factors. Possible reasons for interruption of taking medications were listed with additional open ended option. Questions about interruption of taking medications were asked while participants report missed medications. There were nine knowledge, seven attitude and eight patient provider relationship questions. The correct responses were coded 1 and the incorrect responses coded 0 then the correct answers were added then participants who scored mean and above of the questions were labeled good knowledge, favorable attitude, and good patient-provider relationship (Additional file 1). Internal consistency of the questionnaire found good (Cronbach’s alpha 0.67). The inter-rater reliability was Cohen’s Kappa 0.65. The sensitivity, specificity, and correct classification were 98.3%, 71.2% and 85.0% respectively. Non-adherence was assessed based on number of pills reported to have been actually taken 1 month prior to data collection period divided by number of prescribed pills multiplied by 100%. Patients who missed ≥ 10% of the total prescribed dose were considered non-adherent. Data were collected by four trained BSc nurses through interviewer-administered and reviewing their medical records.

### Data quality control technique

Pretest was conducted among 5% of the sample size before actual data collection and some modification was done. The questionnaire was first prepared in English and translated to local language Amharic and back to English for its consistency. One day training was given for data collectors and supervision was conducted on daily basis throughout data collection.

### Operational definition

#### Non-adherent

Patients who missed ≥ 10% of total prescribed dose were considered non-adherent [10, 16].

#### Knowledgeable, favorable attitude, and good patient-provider relationship

Those respondents who scored points at mean and above for the knowledge, attitude, and patient-provider relationship questions respectively.

#### Alcohol intake

History of alcohol intake since time of starting anti-TB treatment.

#### Comorbidity

Presence of any of chronic disease along with TB.

### Data processing and analysis

Data were checked for its completeness, coded and entered into Epi info Version 7 and exported to SPSS version 20 for analysis. Descriptive statistics were generated including frequency, percent, mean median, and standard deviation (SD). Tables and bar graph were used to display the findings. Univariate logistic regression was used to identify factors associated with non-adherence to tuberculosis treatment. Variables at P ≤ 0.2 in bivariate analysis were taken into multivariate logistic regression model to control possible confounders. Crude odds ratio (COR) and adjusted odds ratio (AOR) with 95% CI were calculated. Variables at P-value < 0.05 in multivariate logistic regression model were considered statistically significant, and odds ratios with corresponding 95% confidence intervals were reported as the measures of the degrees of association.

## Results

### Socio-demographic characteristics of participants

Total of 314 participants were interviewed with 97.5% response rate. The mean age of participants was 35.94 (SD ± 13.83) years. More than half, 166 (54.2%), of participants, were males, 135 (44.1%) single and 193 (63.0%) were orthodox christians. Two-thirds 193 (63.0%) were Amhara by ethnicity. majority, 256 (83.7%), and a quarter, 75 (24.5%), were urban dwellers and grade 9–12 by education, respectively. One hundred forty-five (47.4%) were had distance of 3–5 km from TB clinic and more than half 158 (51.6%) had > 30 min traveling time (Table 1).

**Table 1:** Socio-demographic characteristic of TB patients attending TB clinic in health centers at Gondar town, Northwest Ethiopia, 2017 (n = 306)

Variable	Frequency (n)	Percent (%)
Sex		
Male	166	54.2
Female	140	45.8
Age		
18–28	75	24.5
29–38	61	19.9
39–48	83	27.2
≥ 49	87	28.4
Marital status		
Single	135	44.1
Married	117	38.2
Divorced	36	11.8
Widowed	18	5.9
Religion		
Orthodox	193	63.0
Protestant	58	19.0
Muslim	55	18.0
Ethnicity		
Amhara	234	76.5
Tigrie	40	13.0
Kimant	32	10.5
Residence		
Urban	256	83.7
Rural	50	16.3
Educational status		
Unable to read and write	51	16.7
Able to read and write	65	21.2
Grade 1–8	49	16.0
Grade 9–12	75	24.5
Diploma	27	8.8
Degree and above	39	12.8
Occupational status		
Government employee	119	38.9
Merchant	35	11.4
Farmer	25	8.2
Housewife	47	15.4
Student	27	8.8
Daily laborer	29	9.5
Unemployed	24	7.8
Income (Ethiopian Birr)		
≤ 1000	134	43.8
1001–2000	81	26.5
2001–3000	50	16.3
> 3000	41	13.4
Distance from TB clinic (single trip) (km)		
< 3	70	22.9
3–5	145	47.4
> 5	91	29.7
Type of transportation to the TB clinic		
Walking/foot	84	27.5
Public transport	222	72.5
Traveling time (single trip) (min)		
≤ 30	148	48.4
> 30	158	51.6
Cost of traveling (single trip) (n = 222) (Ethiopian Birr)		
≤ 10 Birr	114	51.4
> 10 Birr	108	48.6

### The overall level of non-adherence to anti-TB therapy

In this study, the rate of non-adherence to anti-TB therapy was 65 (21.2%) (95% CI 17.2, 26.1). The rate is higher (47.0%) among return after default treatment category and lower (19.1%) among new category.

### Participants’ reasons for interruption of taking anti-TB medications

**Fig. 1:**
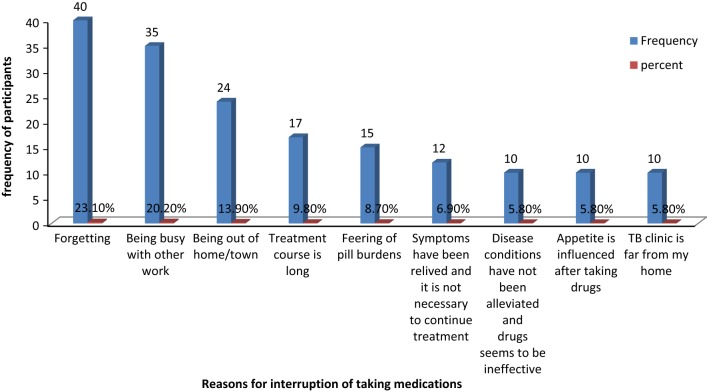
Reasons for interruption of taking anti-TB medications of participants attending TB clinic in health centers at Gondar town, Northwest Ethiopia, 2017 (n = 173)

Participants were asked about reason of interruption of taking medications while they report missing any number of medications. Seventy participants were reported missed anti-TB medications. Most of participants report more than one reason for missing. Forgetting 40 (23.1%), Being busy with other work 35 (20.2%), and being out of home/town 24 (13.9%) were the major reasons of participants for interruption of taking anti-TB medications (Fig. 1).

### Factors associated with non-adherence to anti-TB therapy

In this study; treatment phase, co-morbidity, knowledge, patient-provider relationship, and alcohol intake were significantly associated factors.

Participants who were in continuation phase of treatment were 2.27 times (AOR = 2.27, 95% CI (1.54, 5.94)) more likely non-adhere to their anti-TB therapy than those in intensive phase. Participants with more than one co-morbidity were 6.22 (AOR = 6.22; 95% CI (2.21, 17.48)) more likely to be non-adhere than participants with no or one co-morbidity. Furthermore, participants who had poor knowledge about TB and anti-TB therapy were 4.11 times (AOR = 4.11; 95% CI 1.57, 10.75) more likely to be non-adherent compared with participants with good knowledge. In addition, participants who had poor patient-provider relationship were 4.6 times (AOR = 4.60, 95% CI 1.63, 12.97) as likely be non-adherent as who had good patient-provider relationship. The odds of anti-TB non- adherence was found high among participants who were alcohol intake history (AOR = 5.03; 95% CI 1.54, 16.40) (Table 2).

**Table 2:** Univariate and multivariate analysis for non-adherence to anti-TB therapy among TB patients attending TB clinic in health centers at Gondar town, Northwest Ethiopia, 2017 (n = 306)

Variables	Adherence status		COR (95% CI)	AOR (95% CI)	P-value
	Adherent	Non adherent			
Sex					
Male	133	33	0.84 (0.48, 1.45)		
Female	108	32	1		
Age					
18–28	62	13	1	1	
29–38	48	13	1.29 (0.55, 3.04)	3.22 (0.68, 15.17)	0.139
39–48	70	13	0.89 (0.38, 2.05)	0.83 (0.18, 3.88)	0.808
≥ 49	61	26	2.03 (0.96, 4.32)	2.44 (0.53, 9.41)	0.272
Marital status					
Single	113	22	0.24 (0.09, 0.69)*	0.11 (0.01, 1.60)	0.105
Married	91	26	0.36 (013, 0.99)*	0.09 (0.01, 1.30)	0.077
Divorced	27	9	0.42 (0.13, 1.38)	0.15 (0.01, 3.22)	0.228
Widowed	10	8	1	1	
Residence					
Urban	205	51	0.73 (0.36,1.46)		
Rural	36	14	1		
Educational status					
Unable to read and write	41	18	0.59 (0.16, 2.12)	1.28 (0.21, 7.62)	0.789
Able to read and write	45	20	2.62 (0.96, 7.12)	1.12 (0.20, 6.37)	0.899
Grade 1–8	44	5	1.24 (0.40, 3.80)	0.41 (0.06, 3.01)	0.381
Grade 9–12	65	10	1.01 (0.35, 2.93)	2.12 (0.34, 13.11)	0.420
Diploma	22	5	1.10 (0.30, 4.07)	1.48 (0.19, 11.66)	0.710
Degree and above	32	7	1	1	
Income (Ethiopian Birr)					
≤ 1000	102	32	1	1	
1001–2000	60	21	1.12 (0.59, 2.12)	1.65 (0.50, 5.51)	0.414
2001–3000	43	7	0.52 (0.21, 1.27)	0.47 (0.09, 2.38)	0.365
> 3000	36	5	0.44 (0.16, 1.22)	0.12 (0.01, 1.30)	0.081
Distance from TB clinic (single trip) (km)					
< 3	65	5	1	1	
3–5	121	24	2.58 (0.94, 7.08)	1.83 (0.38, 8.78)	0.450
> 5	55	36	8.51 (3.12, 23.18*	4.30 (0.80, 23.16)	0.090
Type of transportation to the TB clinic					
Walking/foot	72	12	1	1	
Public transport	169	53	1.88 (0.95, 3.73)	1.80 (0.54, 6.01)	0.338
Traveling time (single trip) (min)					
≤ 30	131	17	0.30 (0.16, 0.55)*	0.48 (0.11, 2.11)	0.330
> 30	110	48	1	1	
Cost of traveling (single trip) (n = 222) (Ethiopian Birr)					
≤ 10 Birr	97	17	0.40 (0.21, 0.77)*	1.14 (0.25, 5.24)	0.866
> 10 Birr	75	33	1	1	
Patients category					
New	190	45	1	1	
Treatment failure	18	7	1.64 (0.63, 4.17)	0.52 (0.05, 5.12)	0.58
Relapse	24	5	0.88 (0.32, 2.43)	0.94 (0.19, 4.75)	0.94
Return after default	9	8	3.75 (1.37, 10.27)*	0.88 (0.05, 14.39)	0.93
Treatment phase					
Intensive phase	183	35	1	1	0.030
Continuation phase	58	30	2.70 (1.53, 4.78)*	2.27 (1.5, 5.94)	
HIV status					
Seronegative	153	52	2.30 (1.19, 4.46)*	1.97 (0.41, 9.39)	0.651
Seropositive	88	13	1	1	
TB status disclosure to the family					
Yes	192	45	1	1	
No	49	20	1.74 (0.94, 3.22)	1.47 (0.31, 6.90)	0.626
Number of comorbidity?					
> 1	89	49	5.23 (2.81, 9.74)	6.22 (2.21, 17.48)**	0.001
No or 1	152	16	1	1	
Knowledge					
Good knowledge	156	20	1	1	0.004
Poor knowledge	85	45	4.13 (2.29, 7.44)*	4.11 (1.57, 10.75)**	
Patient-provider relationship					
Good patient-provider relationship	183	32	1	1	0.004
Poor patient-provider relationship	58	33	3.25 (1.84, 5.75)*	4.60 (1.63, 12.97)**	
Alcohol intake					
Yes	35	23	3.22 (1.73, 6.00)*	5.03 (1.54, 16.40)**	0.007
No	206	42	1	1	

*Variables those were significant during univariate logistic analysis at P value 0.05

**Variables that were found to have significant association during multivariate analysis at P-value < 0.05

## Discussion

In this study, the rate of non-adherence to antiretroviral therapy was found 21 point two percent This is in-line with studies done at Arba Minch governmental health institutions [9], Dawouro-zone public healthcare facilities [10], and Mbarara Hospital, Uganda [17] which reported 24.7%, 24.5% and 25%, respectively.

However, it is higher than studies done in North Gondar Zone- Northwest Ethiopia (10% and 13.6%) [16], Khartoum state, Sudan (14%) [18], State of Parana (8.5% %) [19], Kosovo (14.5%) [6], and Thailand (15.6%) [8]. This difference might be due to differences in socio-demographic characteristic, sample size, study designs, settings and time difference.

This finding is lower than studies conducted in Mekele, Ethiopia (55.8%) [20], E ward of Mumbai Municipal Corporation, India (50%) [7], Schenzhen, China (33.74%) [21]. The variation might be due to differences in study settings, study design, and socio-demographic characteristics. Study participants in Mekele were TB/HIV co-infected and those TB patients attending in hospital were included. The study in E ward of Mumbai Municipal Corporation was prospective cohort study and in Schenzhen, China all health facilities with TB treatment service were included.

In the current study participants in the continuation phase of treatment had significant association with non-adherence. Possible justification could be patients in continuation phase might have improved sign and symptoms of disease and expected as they are cured, thus they might be careless in taking medications. This finding is supported by studies in North Gondar Zone-Northwest Ethiopia, Kassala state, Sudan [22], Mbarara Hospital-Uganda. Number of co-morbidity had significant association with outcome variable. Participants who had more than one co-morbidities were had poor adherence to anti-TB therapy, like similar study reported in North Gondar Zone-Northwest Ethiopia, uMgmgundlovu health district.

Poor knowledge about tuberculosis and anti-TB therapy had significant association with non-adherence. This is similar to the results of studies in Dawouro-zone public healthcare facilities, E ward of Mumbai Municipal Corporation-India, Schenzhen-China. Poor patient-provider relationship also had significant association. This agreed to findings of studies in Sodo woreda, Southern Ethiopia [23]. Besides, alcohol intake had significant association with non-adherence. This is similar to the State of Parana, Mbarara, and Baringo, Kenya [24].

Forgetting, Being busy with other work, and being out of home/town were the major reasons for most participants for interruption of taking anti-TB medications. Different studies in North Gondar Zone, Alamata District, Mekele, and Baringo-Kenya, revealed as forgetting was the major reason for medication taking interruption/non-adherence. Being out of home/town was supported by studies in North Gondar Zone and Alamata District.

The finding of this study gives evidence based information for Federal Minister of Health of Ethiopia, regional health office, zonal and district health offices and other stake holders and the information will be used to design TB reduction strategies and take action to further decrease the level of drug non-adherence on anti-TB treatments and improve the outcome of TB treatments.

## Conclusion

This study revealed relatively high non-adherence rate of tuberculosis treatment. To decline the TB treatment non-adherence and to improve treatment outcome of TB-patients; health professionals, health programmers and other stakeholders should give emphasis to prevention of co-morbidities, improving knowledge through health education, providing strong counseling about drug adherence with more emphasis on continuation phase of treatment and about disadvantage of alcohol intake, and strengthening of patient-provider relationship.

## Limitations

Non-adherence was assessed according to data actually taken during the previous 1 month. So, participants might be subjected to recall bias. Patients attending the Hospital and health Posts were not included. This might impose limitation on generalization of findings to all TB patients in the town. In addition, this study did not assess the frequency of missed medications.

## Additional file

**Additional file 1.** Questionnaire. Questionnaire English Version. The objectives of this study is to assess Non-Adherence to Anti-tuberculosis Treatment, Reasons and associated factors among TB patients attending at Gondar town health centers, Northwest Ethiopia . The questionnaire has socio-demographic information, characteristics of tuberculoses and anti- tuberculoses treatment, reasons for interruption taking medications, knowledge and attitude towards tuberculosis and anti-TB treatment, patient-provider relationship, and behavioral factors.

## Abbreviations

AOR: adjusted odds ratio
CI: confidence interval
COR: crude odds ratio
DOTs: directly observed therapies
HIV: human immune deficiency virus
SD: standard deviation
SPSS: statistical package for social sciences
TB: tuberculosis
WHO: World Health Organization
