# Supplementary material for: Non-adherence to anti-tuberculosis treatment, reasons and associated factors among TB patients attending at Gondar town health centers, Northwest Ethiopia
Source: BMC Res Notes. 2018 Oct 1;11:691. doi: 10.1186/s13104-018-3789-4 (PMC6167840; doi:10.1186/s13104-018-3789-4)
Supplement: Supplementary file 1 — Additional file 1. Questionnaire. Questionnaire English Version. The objectives of this study is to assess Non-Adherence to Anti-tuberculosis Treatment, Reasons and associated factors among TB patients attending at Gondar town health centers, Northwest Ethiopia . The questionnaire has socio-demographic information, characteristics of tuberculoses and anti- tuberculoses treatment, reasons for interruption taking medications, knowledge and attitude towards tuberculosis and anti-TB treatment, patient-provider relationship, and behavioral factors. [file 13104_2018_3789_MOESM1_ESM.docx]

### Questionnaire English Version

Hello, my name is________ and I am going to conduct an interview with you on behalf of __________.You are just invited to participate in a research study to be conducted by __________ at _______________.

Thank you for allowing us to share your precious time and for your willingness to participate in this study. The objectives of this study is to assess **Non-Adherence to Anti-tuberculosis Treatment, Reasons and associated factors among TB patients attending at Gondar town health centers, Northwest Ethiopia** and you are chosen to participate in this study.

In order to attain effectively the goal, we are asking you for your generous help. We would like to assure you that privacy will be strictly maintained throughout. There is no need to put your name on the format. No individual response will be reported. It is your full right to participate or refuse in the study. If you don’t want to participate in the study you have full right to refuse. But your honest participation will have a great contribution. So please take a few minute to answer these questions.

Do you wish to participate in the study?

………Yes, I want to participate in the study (please go to the next page)

……….No, I don’t want to participate

Thank you

- Questionnaire number___________________ signature _______________

**Part I: Socio-demographic information**

| **No** | **Questions** | **Coding Categories** | **Code** |
| --- | --- | --- | --- |
| 101 | Sex | 1. Male 2.Female |  |
| 102 | Age | ___________ Years. |  |
| 103 | Marital Status | 1. Single  2. Married  3. Divorced  4. Widowed |  |
| 104 | Religious affiliation | 1. Orthodox  2 Muslim  3. Protestant  4. Catholic  5. Others specify _____ |  |
| 105 | Ethnic group | 1. Amhara  2. Oromo  3. Tigirie  4. Kimant  5.Others specify ______ |  |
| 106 | Residence | 1. Urban 2. Rural |  |
| 107 | Education Status | 1. Unable to read and write  2. Able to read and write  3. Grade 1 -8 4. Grade 9-12  5. Certificate 6. Diploma  7.. Degree and above |  |
| 108 | Family’s monthly income (birr) | _______________ birr |  |
| 109 | Occupation | 1. Government 2. Merchant 3. Farmer 4. Housewife 5. Student 6. Non employed 7. Retired 8. Other(please specify:_______ |  |
| 110 | Distance from residence house to DOT center | -------- KM ---------meter |  |
| 111 | Travelling time | ________hour _______minute |  |
| 112 | Type of transportation | 1. Walking 2. Use own transport 3. Use public transport |  |
| 113 | Cost of travel for a single trip in ETB | ___________ ETB |  |

**Part II: Characteristics of tuberculoses and anti- tuberculoses treatment**

| No | Questions | Coding Categories | Code |
| --- | --- | --- | --- |
| 201. | Disease classification | 1. Pulmonary TB positive 2. Pulmonary TB negative 3. Extra pulmonary TB 4. MDR TB |  |
| 202 | Patients category | 1. New 2. Treatment failure 3. Relapse 4. Return after default |  |
| 203 | Treatment phase | 1. Intensive phase 2. Continuation phase |  |
| 204 | Treatment regimen | 1. New patient treatment regimen 2. Retreatment patient regimen |  |
| 205 | HIV status | 1. Sero-negative 2. Sero- positive 3. Unspecified |  |
| 206 | ART status | 1. Started 2. Not started |  |
| 207 | Duration since the diagnosis of TB | ________month/Years |  |
| 208 | Duration since you starting anti-TB treatments? | ________month /years |  |
| 209 | TB status disclosure to the family | 1. Yes 2. No |  |
| 210 | No. of Antihypertensive drugs? | 1. Mono-therapy 2. Two drugs 3. Three drugs or more |  |
| 211 | How many anti TB tablets prescribed during the previous one month? | ____________ |  |
| 212 | How many anti TB tablets do you miss during the previous one month? | _____________ |  |
| 213 | How many anti TB tablets do you have now? | ______________ |  |
| 214 | Number of tablets per day? | ---------------------- |  |
| 215 | Frequency of Daily doses? | 1. Once Daily 2. Two times a day (BID) 3. Three times a day or more (>=TID) |  |
| 216 | Do you have co -morbidity? | 1. Yes 2. No |  |
| 217 | If your answer is yes for Number 317, what is it? | ___________________ |  |

**Part III: Reasons for interruption of taking medications**

1. Symptoms have been relieved and it is not necessary to continue treatment

2. Disease conditions have not been alleviated after treatment and the drugs seem to be ineffective

3. Treatment is not necessary as I am so old

4. I am working busy

5. Treatment course is too long and the dose is too large

6. I always forget to take drugs

7. Adverse drug reactions are severe

8. I worry about my body damaged by anti-tuberculosis drugs

9. Appetite is influenced after taking drugs

10. Other diseases cause interruption

11. Migration

12. Following doctor's advices

13. Following other's suggestions

14. I am not satisfied with health-care services

15. Financial difficulty and higher medical cost

16. Other reasons

**Part IV: Knowledge towards Tuberculosis and anti-TB treatment**

| **No** | **Questions** | | **Coding Categories** | **Code** |
| --- | --- | --- | --- | --- |
| 401 | What is Tuberculosis? | | 1. A terrible headache with dizziness  2. A harmless cough  3. A passing diarrhea  4. A dangerous lung disease  5. I don’t know |  |
| 402 | What are signs of tuberculosis? | | 1. Coughing and difficulty breathing  2. Tiredness, fever  3. Coughing of blood, weight loss, fever, tiredness  4. Diarrhea, fever, weight loss  5. I don’t know |  |
| 403 | What is the cause of tuberculosis? | | 1. A dry period with strong sun  2. The cause is god’s punishment  3. A very long coughing period  4. A bacterium  5. I don’t know |  |
| 404 | How does tuberculosis spread? | | 1. By air  2. By air and by milk from infected cattle  3. By water and milk  4. It does not spread  5. I don’t know |  |
| 405 | Is tuberculosis treatable? | | 1. Yes, at medical clinics with injections  2. Yes, the local healer can treat it  3. Yes, at a medical clinic with pills for a long time 4. No  5. I don’t know |  |
| 406 | When is tuberculosis cured? | | 1. When all coughing and fever are gone  2. When therapy is completed (all pills taken)  3. When the person feels good again  4. When the person regains his weight  5. I don’t know |  |
| 407 | How can tuberculosis be prevented? | | 1. Good ventilation and boiling of milk  2. By eating well  3. By having a strong health  4. By vaccination  5. I don’t know |  |
| 408 | Should all pills be taken? | | 1. No, this is useless  2. No, this is harmful  3. Yes, this is important  4. Yes, but only if the patient is sick  5. I don’t know |  |
| 409 | Treatment time | | 1. < 6 months  2. 6 months  3. > 6 months  4. Do not know |  |
| **Part V: Attitude towards tuberculosis and anti- TB treatment** | | | | |
| **No** | **Questions** | **Coding Categories** | | **Code** |
| 501 | Concern about contracting TB | 1. A lot  2. A little  3. Not concerned | |  |
| 502 | What is your belief about the extent of importance of anti- tuberculosis drugs used for TB treatment? | 1. Important 2. Not important | |  |
| 503 | Choices of care for Tb | 1. Health facilities  2. Pharmacy  3. Traditional healer  4. Pursue self treatment option | |  |
| 504 | Do you think you can get TB after cured with this treatment? | 1. Yes 2. No 3. I don’t know | |  |
| 505 | What is your feeling about TB | 1. Feel ashamed 2. Don’t feel ashamed | |  |
| 506 | Do you feel as waiting time is Long | 1. Yes 2. No | |  |
| 507 | Do you feel as distance of the OTC center is long | 1. Yes 2. No | |  |

**Part VI: Patient-provider Relationship**

| No | Questions | Coding Categories | Code |
| --- | --- | --- | --- |
| 601 | Are care providers greets you well? | 1. Yes 2. No |  |
| 602 | Do you get counseling and education on the disease and its treatment? | 1. Yes 2. No |  |
| 603 | Are they giving you adequate contact time? | 1. Yes 2. No |  |
| 604 | Are they treating you equally? | 1. Yes 2. No |  |
| 605 | Are they motivates or encourages you in the treatment activities? | 1. Yes 2. No |  |
| 606 | Are they kept confidentiality regarding all the information that you shared to providers? | 1. Yes 2. No |  |
| 607 | Are they kind and Polite? | 1. Yes 2. No |  |
| 608 | Do you get the necessary respect from care providers? | 1. Yes 2. No |  |

Part VII: **Behavioural factors**

1. Do you smoke?
2. Yes
3. No
4. Do you drink alcohol?
5. Yes
6. No
